# Interleukin-6 at the Host-Tumor Interface: STAT3 in Biomolecular Condensates in Cancer Cells

**DOI:** 10.3390/cells11071164

**Published:** 2022-03-30

**Authors:** Pravin B. Sehgal

**Affiliations:** 1 Department of Cell Biology & Anatomy, New York Medical College, Valhalla, NY 10595, USA; pravin_sehgal@nymc.edu; Tel.: +1-914-594-4196; 2 Department of Medicine, New York Medical College, Valhalla, NY 10595, USA

**Keywords:** cytokines, interleukin-6 (IL-6), cancer cells, stromal cells, macrophages, epithelial to mesenchymal transformation (EMT), sex bias, p53 mutations, STAT3 signaling, transcriptional regulation, biomolecular condensates, discrepant data for IL-6 in the human circulation, chaperone (enhancing) effects of anti-IL-6 antibodies, immunotherapy

## Abstract

It was recognized over 30 years ago that the polyfunctional cytokine interleukin-6 (IL-6) was an almost invariant presence at the host-tumor interface. The IL-6 in the tumor microenvironment was produced either by the cancer cell or by host stromal cells, or by tumor-infiltrating immune cells, or all of them. IL-6 effects in this context included local changes in tumor cell-cell and cell-substrate adhesion, enhanced motility, epithelial to mesenchymal transformation (EMT), and changes in cell proliferation rates in both solid tumors as well as hematologic dyscrasias. Locally produced IL-6 enhanced cancer-targeting functions of tumor-infiltrating macrophages and immune cells. Additionally, the sex-biased phenotype of certain cancers [e.g., hepatocellular carcinoma (HCC) which is 3-5-fold more common in men] was related to the inhibition of macrophage-derived IL-6 production by estradiol-17β (E2). In many circumstances, locally produced IL-6 reached the peripheral circulation and elicited systemic effects such as cachexia and paraneoplastic syndrome (including fever, increased erythrocyte sedimentation rate, increased levels of C-reactive protein in serum, hypoalbuminemia). This review highlights the EMT produced by IL-6 in cancer cells, as well as mechanisms underlying sex bias in HCC, enhanced IL-6 expression in cancer cells resulting from mutations in p53, consequent alterations in STAT3 transcriptional signaling, and the newer understanding of STAT3 nuclear bodies in the cancer cell as phase-separated biomolecular condensates and membraneless organelles (MLOs). Moreover, the perplexing issue of discrepant measurements of IL-6 in human circulation using different assays, especially in patients undergoing immunotherapy, is discussed. Additionally, the paradoxical chaperone (enhancing) effect of anti-IL-6 “neutralizing” antibodies on IL-6 in vivo and consequent limitations of immunotherapy using anti-IL-6 mAb is considered.

## 1. Introduction

Soon after the discovery of the polyfunctional cytokine interleukin-6 (IL-6) in the early 1980s it became apparent that this cytokine was readily expressed in and secreted by a variety of cell types in response to a large number of stimuli, including viral and bacterial infections, sterile inflammation, tissue injury, and in response to other cytokines and growth factors including interleukin-1, tumor necrosis factor and platelet-derived growth factor (reviewed in [1]). An early study reported that many human solid tumors were immunoreactive for IL-6 [2]. In different tumors, this IL-6 was associated with the neoplastic cell elements, stromal cells, or both. This tumor-localized IL-6 is now thought to have effects on tumor cells, including activation of pro-oncogenic STAT3 signaling, enhancement of cell motility, reduction in cell-cell adhesion, promotion of epithelial to mesenchymal transformation (EMT), enhanced cell proliferation and thus, overall, enhanced metastatic spread [3,4,5,6,7]. Moreover, IL-6, in its capacity as a B-cell growth and differentiation factor, was implicated as a driver of neoplastic cell proliferation in hematopoietic dyscrasias including multiple myeloma and Castleman’s disease and other hypergammaglobulinemic conditions such as cardiac myxoma [8,9,10,11]. Patients with a variety of solid tumors, as well as mice bearing experimental solid tumors, exhibited elevated levels of IL-6 in the circulation [8,9,10,11,12]. This systemic IL-6 spillover is considered a basis for paraneoplastic syndrome, which includes cachexia (loss of body weight), fever, enhanced erythrocyte sedimentation rate, and altered acute phase plasma protein levels (e.g., enhanced C-reactive protein levels and hypoalbuminemia) [8,9,10,11,12,13,14,15,16,17]. 

Secreted human IL-6 corresponds to differentially modified 184-amino acid proteins derived from a single gene located at 7p15–21 [1,11]. The secreted IL-6 proteins are differentially O- and N-glycosylated, giving rise to multiple modified proteins in the size range of 21–30 kDa [18,19,20,21]. There is cell-type specificity in these modifications, with certain cell lines secreting the mainly nonglycosylated 21 kDa protein [20]. This IL-6 heterogeneity is also reflected in the detection of multiple IL-6 species in human circulation and body fluids [19]. IL-6 is a member of a family of IL-6 type cytokines which use the common gp130 β chain for signaling to the cell interior; this family includes interleukin-11, leukemia inhibitory factor, oncostatin M, ciliary neurotrophic factor, cardiotrophin-1, novel neurotrophin-1 (collectively “IL-6-type cytokines”; [1,11,17]). The IL-6 receptor on the surface of cells comprises the cytokine-binding 80 kDa α chain (the IL-6Rα) and the common signal transducer 130 kDa β chain (the gp130) [11,17]. While IL-6Rα has a restricted distribution in different cells, gp130 is present more ubiquitously [11,17]. “Classical” IL-6 signaling involves the binding of IL-6 to IL-6Rα associated with the plasma membrane and the binding of this binary complex to the membrane-bound gp130 [11,17]. However, IL-6Rα in its soluble form can also bind to IL-6; this soluble binary complex can also initiate signaling by binding to gp130 at the plasma membrane of a large array of different cells (“trans signaling”; [11,17]). This gp130 also occurs in a soluble form, and IL-6 species in the circulation can form ternary complexes with soluble IL-6R and soluble gp130 fragments; such complexes are typically considered inactive [11,17,22,23]. IL-6 in the circulation can also form complexes with other proteins such as C-reactive protein and albumin [22,23]. This complexity of modifications and protein interactions likely accounts for the difficulties encountered when evaluating IL-6 levels and biological activity in human circulation (see below).

IL-6 gene expression is readily upregulated in cells in response to a wide variety of stimuli (viral and bacterial infections, inflammation and tissue injury, and other cytokines and growth factors) (reviewed in [1,11,24]). At the molecular level, the IL-6 promoter is activated through multiple signaling pathways, including activation of the protein kinase A, protein kinase C, and the NF-κB pathways through a super-enhancer region (“multiple response element,” MRE) of 100–200 nucleotides upstream of the RNA start site [24,25,26]. Conversely, IL-6 expression is repressed by glucocorticoids such as dexamethasone and by estradiol-17β (E2) through mechanisms that include the binding of activated GR to the MRE region and the binding of GR and ER to the p65 subunit of NF-κB [26,27,28,29,30,31]. Indeed, the ability of glucocorticoids to efficiently repress IL-6 gene expression is the basis for the use of glucocorticoids to suppress symptomatic COVID-19 disease today [32]. Moreover, the sex bias seen in the greater severity of COVID-19 symptoms in men [33] may be related to the ability of endogenous E2 in women to reduce IL-6 gene expression. 

In carcinogenesis, the ability of E2 to inhibit MyD88-driven IL-6 production by macrophages has been implicated in the greater prevalence of hepatocellular carcinoma in men than women ([34]; see below). In the context of the host-tumor interface, increased IL-6 production by stromal cells and tumor-infiltrating immune cells (e.g., macrophages) has been implicated as part of the senescence-associated secretory phenotype (SASP) [35,36]. Increased IL-6 production by neoplastic cells has been implicated in pro-oncogenic gene mutations in cancer cells that enhance IL-6 gene expression and secretion [37,38]. Certain cancer cell lines (such as the T24 bladder carcinoma) constitutively produce high levels of IL-6 [39,40]. Additionally, hematopoietic cell types responding to IL-6 with enhanced proliferation are typically observed in diseases such as multiple myeloma and Castleman’s disease [8,9,10,11]. Thus, the dysregulated/enhanced IL-6 gene expression in neoplastic, stromal, or tumor-adjacent immune cells and IL-6-driven cell proliferation contribute to increased aggressiveness of certain neoplastic diseases. 

This review highlights studies of epithelial-mesenchymal transformation (EMT) produced by IL-6 in breast carcinoma cells, as well as mechanisms underlying the contribution of IL-6 to sex bias in liver cancer, the dysregulation of IL-6 promoter function in cancer cells resulting from mutations in p53, alterations in STAT3 transcriptional signaling, and the newer understanding of STAT3 nuclear bodies in the cancer cell as metastable biomolecular condensates. The perplexing issue of discrepant measurements of IL-6 in human circulation using different assays, especially in patients undergoing immunotherapy, is considered. Additionally, the paradoxical chaperone (enhancing) effect of anti-IL-6 “neutralizing” antibodies on IL-6 in vivo and consequent limitations of immunotherapy with anti-IL-6 mAb are discussed. 

## 2. IL-6 Triggers Enhanced Cell Motility and an Epithelial to Mesenchymal Transformation (EMT) in Breast Cancer Cells

In 1989 Tabibzadeh and colleagues reported that IL-6 immunoreactivity was associated with the neoplastic cell elements and stromal cells of most human tumors evaluated [2]. These included adenocarcinomas of mammary, colonic, ovarian, and endometrial origins and adenocarcinomas metastatic to lymph nodes [2]. Additionally, Krueger and colleagues discovered that IL-6 enhanced the proliferation of normal human keratinocytes in cell culture in a manner comparable to that of keratinocyte growth factor [41]. An increase in IL-6 was observed in psoriasis lesions—a skin condition characterized by keratinocyte proliferation [42]. These early observations prompted investigations of the effects of IL-6 on neoplastic cells in solid tumors. Thus, Tamm and colleagues asked whether IL-6 stimulated the proliferation of breast cancer cells T47D and ZR-75-1 and/or affected the cancer-cell phenotype [3,4,5,43,44,45,46].

**Figure 1:**
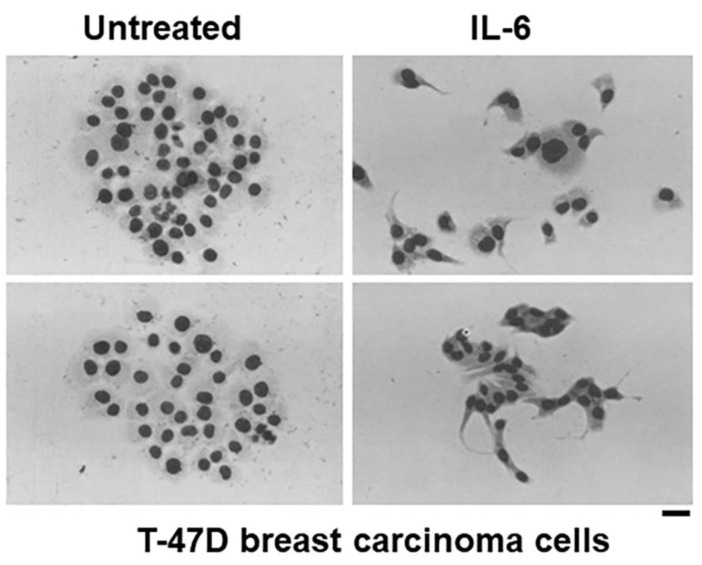
IL-6 increases motility of T-47D ductal breast carcinoma cells. T-47D cells plated at a low density (42 cells/cm^2^ in T-25 flasks) were either left untreated or exposed to rIL-6 (150 ng/mL) for 11 days as indicated. Two representative colonies (Giemsa-stained) under each experimental condition are illustrated. Scale bar = 20 µm. Adapted from Tamm et al., 1989 [3].

**Figure 2:**
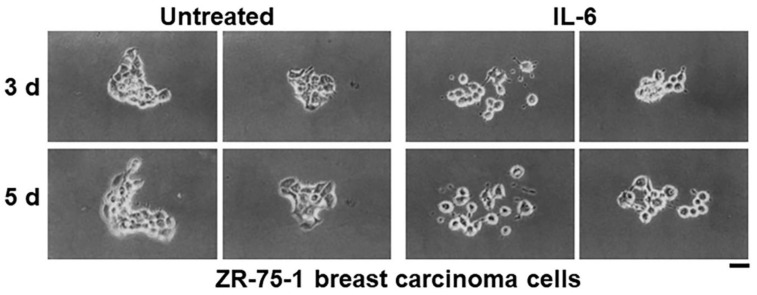
IL-6 increases motility of ZR-75-1 breast carcinoma cells. ZR-75-1 cells plated at a low density (42 cells/cm^2^ in T-25 flasks) were either left untreated or exposed to rIL-6 (15 ng/mL). Live cell images were of the same colonies at 3 or 5 days after plating. Two representative colonies under each experimental condition (at 3 d and 5 d) are illustrated. Scale bar = 30 µm. Adapted from Tamm et al., 1989 [3].

**Figure 3:**
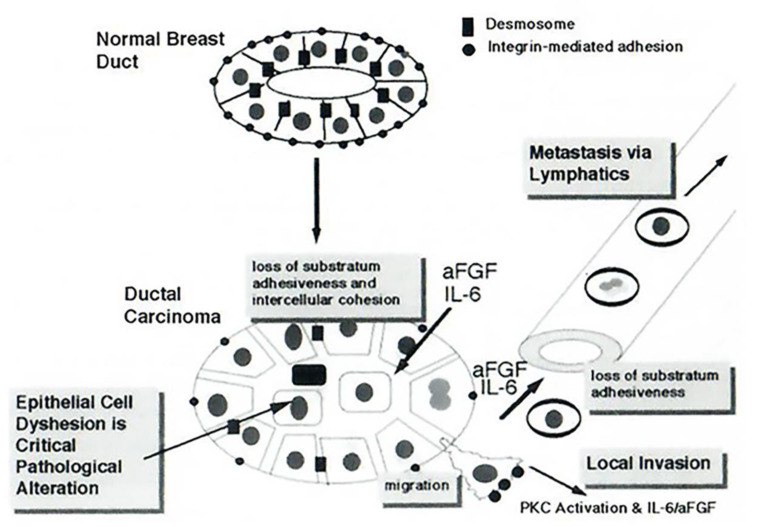
Schematic of cytokine-induced effects on invasiveness and metastasis of breast carcinoma cells. Reprinted with permission from ref. [4]. 1998, Birkhauser Verlag.

In pioneering studies using time-lapse cinemicrography extending for 7–10 days per experiment carried out between 1988 and 1995, Tamm and colleagues discovered that IL-6 had variable effects on the proliferation of breast cancer cells (enhance, inhibit, or no effect depending on cell clones investigated), but unexpectedly, caused a marked increase in breast cancer cell motility and dispersion of cells [3,4,43,44,45,46] (Figure 1 and Figure 2). This increase in cell motility was accompanied by a decrease in focal adhesions between the cells and the substrate, a reduction in the cell cytoskeleton, and a decrease in cell surface E-cadherin [3,4,43,44,45,46]. It was suggested that the cell-cell and cell-substrate dyshesion phenotype produced by IL-6 might contribute to an increased metastatic potential of breast cancer [4] (Figure 3). Thus, IL-6 by itself, and synergistically with acidic fibroblast growth factor (aFGF), could dramatically alter the social behavior of breast cancer cells to enhance cell motility and cancer-cell dispersal in a direction conducive to increased local invasiveness as well as distant metastasis [4] (Figure 3). In brief, in T-47D and ZR-75-1 breast carcinoma cells, the changes triggered by IL-6 comprised changes typically observed in cells undergoing epithelial to mesenchymal (fibroblastoid) transformation (EMT); these included the development of elongated cells with enhanced motility, increased cell-cell separation, decreased adherence type junctions, loss of vinculin and desmoplakin I/II, decreased actin stress fibers, perinuclear retraction of cytokeratin filament connections and cytokeratins as well as reduced cell surface E-cadherin [3,4,43,44,45,46]. Moreover, cells exposed to both IL-6 and aFGF displayed a rounded-cell phenotype but continued to replicate—such cells did not require substrate attachment for proliferation and dissemination [4]. These phenotypic changes were independent of the effects of IL-6 on the proliferation of different clones of breast cancer cells (inhibit, enhance, or no effect in a clone-specific manner) [4].

Observations by Tamm and colleagues were confirmed by Revel and colleagues in T47D, MCF-7, and SK-BR-3 [47] and by Asgeirsson et al. [48] in T-47D, ZR-75-1, and MDA-MB-231 human breast carcinoma cell lines who also emphasized that different cell lines and cell clones displayed different IL-6 responsive phenotypes. Sullivan et al. [49] confirmed the induction of an EMT by IL-6 in MCF-7 breast cancer cells using a 3-D culture system and related these changes to the activation of Tyr-P-STAT3. Walter et al. [50] used an unbiased open-ended approach to isolate the protein factor secreted by human adipose stromal cells, which promoted migration and invasion of breast cancer cells in cell culture and discovered IL-6. Additionally, Walter et al. confirmed that IL-6 enhanced the invasiveness of human breast cancer cells in a mouse xenograft model in which the cancer cells were implanted deep within the renal capsule [50]. 

The ability of IL-6 to trigger an EMT and enhance cancer cell motility has been extended to colorectal cancer, cancer stem cells in blood vessels in head and neck squamous cell carcinomas, hepatocellular cancer, and laryngeal squamous cell carcinoma [51,52,53,54]. The ability of IL-6/STAT3 to modulate additional tumor-promoting and tumor-suppressing pathways have been extensively investigated [11,51,55,56,57,58,59]. These include extensive investigations of the ability to promote the growth of hematopoietic neoplasms [1,8,9,10,11,17]. Numerous recent studies have implicated the ability of IL-6/STAT3 to influence signaling pathways involving Fra-1, SNAIL1, Slug, Twist2 pathways in enhancing the EMT phenotype [51,55,56,57,58,59], culminating in the increase or decrease of the expression of genes collectively affected by activated STAT3 (the “STAT3 signature”). The reader is referred to recent publications such as [59] for this discussion, as well as for the identification of molecules such as ANGPTL4, MMP13, and STC1, which contribute to the enhancement of the EMT phenotype and additional pathways involved (see Figure 7 in [59] for one example).

In as much as many recent publications (e.g., [49,50,51]) fail to cite the pioneering discovery of Dr. Igor Tamm [3,4]) (Figure 1, Figure 2 and Figure 3) in this area, this essay is focused on highlighting the work from the Tamm laboratory. Indeed, Felcher et al. [7] in 2022 state as follows: “The role of IL-6 as a promoter of malignancy in breast cancer has been well established in different models and conditions. In 1989, a report was published demonstrating for the first time (emphasis added) that IL-6 addition to cell culture medium enhanced motility as well as the transition from cuboidal to fibroblastoid-like morphology of ER+ breast cancer cells. Importantly, these effects reverted upon cytokine removal [49].” ([49] in the preceding sentence corresponds to [3] Tamm et al., 1989 in the present review). 

## 3. IL-6 as a Sex-Specific Determinant in Hepatocellular Carcinoma

Hepatocellular carcinoma (HCC) is 3–5 times more prevalent in men than in women; chemical toxicity such as due to alcohol contributes to HCC [34,60,61]. Moreover, experimental HCC in mice treated with carcinogens such as diethylnitrosamine (DEN), or carbon tetrachloride, is more prevalent in male than female mice [34,60]. Karin and colleagues obtained the remarkable insight that it was sex-biased enhanced production of IL-6 by macrophages (Kupfer cells) in the chemically damaged liver, which drove HCC development in males [34]. 

By way of background, first, IL-6 has a growth-promoting effect on hepatocytes in vivo [61]. This is best seen in the manner in which IL-6 promotes hepatic regeneration after partial hepatectomy in a mouse model. IL-6^−/−^ mice show reduced liver regeneration [61]. Second, estradiol-17β (E2) suppresses IL-6 expression in different cell types by inhibiting NF-κB activation [28,29,30,31]. 

To understand why men have a 3-5-fold higher prevalence of HCC than women, Karin and colleagues turned to an experimental model of HCC in mice [34]. In this model, mice administered hepatotoxic DEN developed HCC with a sex bias—HCC was more common in male than female mice. This sex-biased liver carcinogenesis was driven by IL-6 in that IL-6 knockout mice did not show this sex bias. Moreover, male mice administered E2 together with DEN showed reduced HCC development. Furthermore, the cancer-causing mechanism was linked to the MyD88-dependent stimulation of IL-6 production by Kupfer cells (macrophages) in the chemically damaged liver by observing that the sex bias in HCC development was abrogated in MyD88^−/−^ mice. Overall, these studies provided a clearcut example of how sex-biased IL-6 production by tissue-infiltrating macrophages in the tumor microenvironment can drive carcinogenesis [34,60,61]. 

## 4. p53 Mutants Enhance IL-6 Expression and Change Responsiveness in Cancer Cells

Because neoplastic elements in human tumors were immunoreactive to IL-6 antibodies and several cancer cell lines constitutively secreted IL-6 [39,40], we investigated whether cancer-associated genetic changes might upregulate IL-6 gene expression. In as much as mutations in the transcription factor p53 are among the most common alterations in neoplastic cells, we investigated whether p53 affected IL-6 promoter activation and whether mutations in p53 caused dysregulated and aberrant IL-6 gene expression. In transient transfection experiments, we discovered that wild-type (wt) p53 was a strong repressor of the human IL-6 promoter [37,38]. Moreover, p53 mutants isolated from spontaneous solid tumors had not only lost the ability to repress IL-6 expression but had gained the ability to enhance activation (a “gain of function” mutation) [37,38]. These data were consistent with the hypothesis that enhanced IL-6 production by cancer cells might be a consequence of cancer-associated mutations in p53. This hypothesis was strengthened by the observation that the p53Val^135^ temperature-sensitive mutant was able to repress the IL-6 promoter at the wt temperature (32.5 °C) but enhanced IL-6 expression at the mutant temperature (37 °C) [62]. 

Moreover, p53 also affected the responsiveness of cancer cells to IL-6 by modulating both the C/EBPβ (NF-IL6) and STAT3 pathways [63,64]. Wt p53 reduced the ability of IL-6 to enhance the synthesis and secretion of fibrinogen and α_1_-antichymytrypsinogen (ACT) (a pathway requiring C/EBP transcription factors) [58]; the cancer-derived mutants of p53 lost this ability. Similarly, wt p53 inhibited IL-6-induced STAT3 signaling, but cancer-derived mutants had lost this ability [64]. The p53Val^135^ ts mutant displayed the relevant phenotype at the wt and mutant temperatures confirming the involvement of p53 in IL-6-induced mechanisms [62].

In a novel observation, Hep3B hepatocytes expressing wt p53 exposed to IL-6 displayed a marked loss of immunostaining for STAT3 even though the STAT3 protein was intact in the cells as assayed by Western blotting [64]. This phenomenon, dubbed “STAT-masking,” has now been interpreted to represent a phase transition of STAT3 in the cytoplasm to a form of biomolecular condensate structures inaccessible to anti-STAT3 antibody [65]. This phase transition involved a not yet identified wt p53-induced cellular protein, IL-6-induced Tyr-phosphorylation of STAT3, was transient (lasted 2–3 h) and was blocked by the proteasomal inhibitors lactacystin and MG-132. The Hep3B cell expression mutant p53 species did not display a STAT3 masking phenotype in response to the IL-6 effect. The physical mechanisms underlying phase transitions observed in STAT masking remain poorly understood. 

In exciting new observations, Cheteh et al. report that IL-6 produced by cancer-associated fibroblasts attenuate the p53-driven proapoptotic response elicited by doxorubicin in prostate cancer cells [66]. Under these circumstances, stromal-derived IL-6 in the tumor microenvironment would enhance development of resistance to chemotherapy [66]. 

## 5. Phase-Separated STAT3 Cytoplasmic and Nuclear Bodies

**Figure 4:**
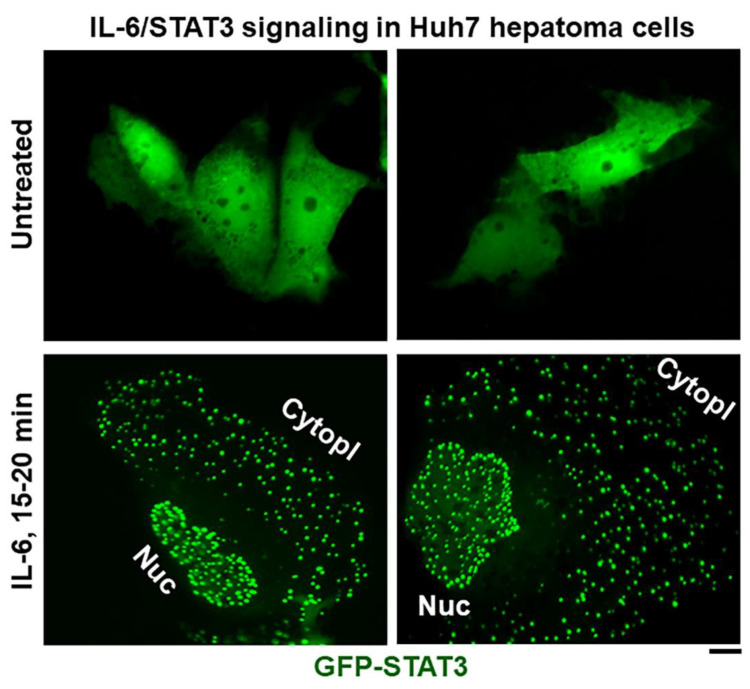
IL-6-induced cytoplasmic and nuclear GFP-STAT3 bodies (“biomolecular condensates”) in Huh7 hepatoma cells. Sub-confluent cultures of Huh7 cells in 35 mm plates that had been transiently transfected with the pGFP-STAT3 expression vector one day earlier were imaged using live-cell microscopy without IL-6 exposure or after exposure to IL-6 (20 ng/mL) for 15–20 min Nuc–nucleus, Cytopl–cytoplasm. Scale bar = 25 μm. Adapted from Sehgal, 2019 [65].

**Figure 5:**
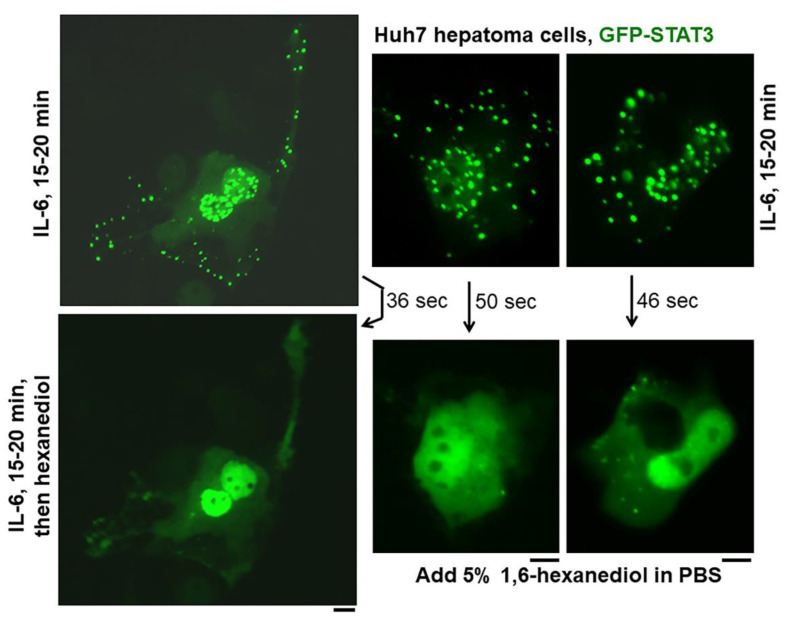
IL-6-induced cytoplasmic and nuclear GFP-STAT3 bodies in Huh7 hepatoma cells are phase-separated biomolecular condensates (also called “membraneless organelles” or MLOs). Huh7 cultures in 35 mm plates transfected with pGFP-STAT3 vector one day earlier were treated with IL-6 (20 ng/mL) for 15–20 min. Live cells showing IL-6-induced cytoplasmic and nuclear bodies at 15–20 min were identified, then exposed to hexanediol (5%) in phosphate-buffered saline (PBS) and imaged immediately thereafter as indicated. Figure illustrates three independent experiments. Scale bar = 25 μm. Adapted from Sehgal, 2019 [65].

It is now widely accepted that STAT3 activation in neoplastic cells through Tyr- and/or Ser-phosphorylation (by paracrine IL-6 or intrinsic activation) [6] contributes dramatically to the pro-oncogenic phenotype through enhanced/altered transcriptional activity of STAT3 in the nucleus. This has been referred to as the “STAT3 signature” (see Figure 7 in [59] and citations therein). What is less appreciated is that the nuclear transcriptional effects of activated STAT3 take place at the level of “nuclear bodies,” which are phase-separated liquid-liquid droplets comprising activated STAT3 together with proteins such as CREB-binding protein (CBP) and acetylated histone H4, which are markers for transcriptionally active chromatin [65,67,68]. The formation of STAT3 nuclear bodies required Tyr-phosphorylation, and in fluorescence recovery after photobleaching (FRAP) assays, the GFP-STAT3 is readily exchangeable in the nuclear compartment [65,67,68]. Thus, STAT3 transcriptional activity in cancer cells occurs at the level of IL-6-induced nanodroplets, which comprise phase-separated biomolecular condensates [65,68] (Figure 4). As a test for their phase-separated nature, these STAT3 nuclear droplets can be disrupted by 1,6-hexanediol and hypotonic stress [65,68] (Figure 5). Moreover, IL-6-activated STAT3 condensates have also been observed in the cytoplasm of hepatoma cells; these are disrupted by 1,6-hexanediol and hypotonic stress [65] (Figure 4 and Figure 5). These observations reflect a new area of science relating to biomolecular condensates formed by activated STAT3 in the cancer cell [68]. Such STAT3 phase-separated condensates may provide new targets for interfering with the pro-oncogenic consequences of this transcription factor. Indeed, cancer-promoting proteins, such as the EML4-ALK fusion protein, appear to activate STAT3 in the nucleus by novel interactions with STAT3 at the level of such phase-separated nanodroplets [69]. 

## 6. Discrepant Measurements of IL-6 in the Human Circulation

**Figure 6:**
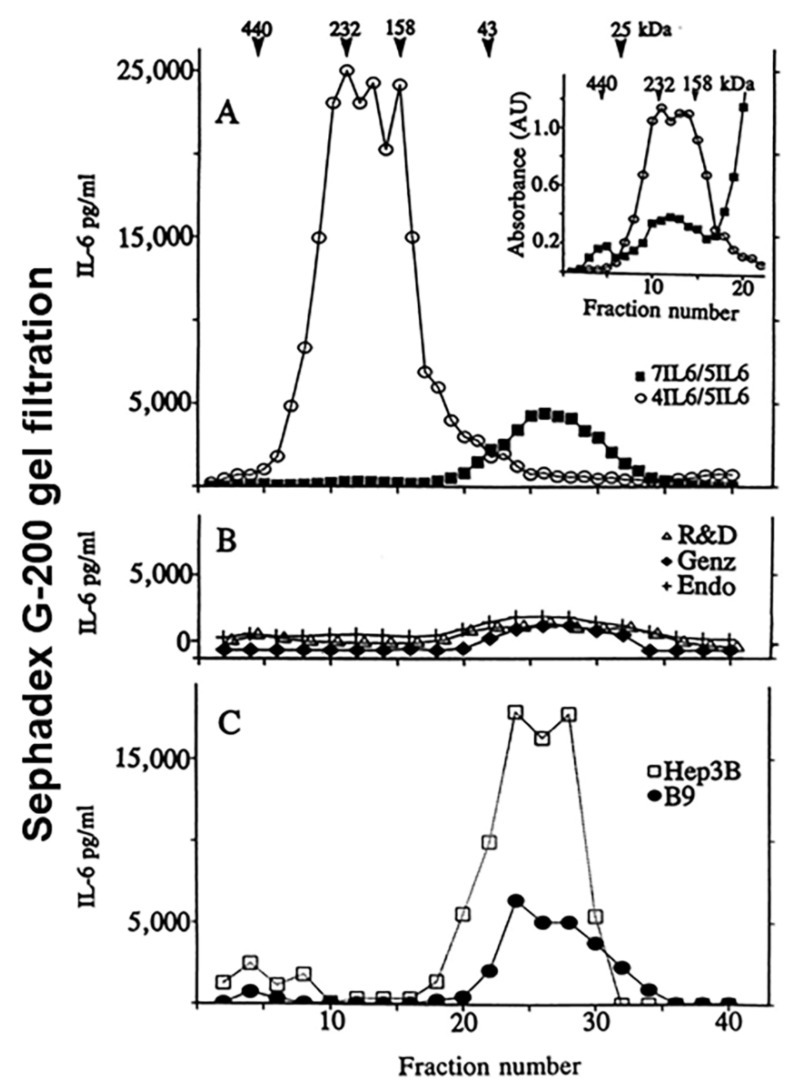
Distinct classes of chaperoned IL-6 in human blood–differential immunological and biological availability. Sephadex G-200 gel filtration and immunologic and biologic characterization of IL-6 complexes in serum from a melanoma patient who was first actively immunized using the received mAb-KLH+BCG regimen and then using an autologous anti-cancer antigen preparation (as in [24,67]). One ml of serum was fractionated by gel filtration and the eluate fractions were assayed for IL-6 in five different ELISAs and two bioassays. Panel A, IL-6 concentrations assayed in the 7IL6/5IL6 (▪) and 4IL6/5IL6 (o) ELISAs. The inset illustrates the absorbance values recorded in each of these two ELISAs using the indicated elution fractions. Panel B, IL-6 concentrations assayed using three different commercial kits and expressed in terms of the kit standards: R&D (Δ), Genzyme (♦), and Endogen (+). Panel C. IL-6 concentrations assayed using the B9 hybridoma proliferation (●) or the hepatocyte Hep3B stimulation bioassays (□). Adapted from Ndubuisi et al., 1998 [70].

Since 1989 we have been faced with a conundrum. Hep3B hepatocytes respond to IL-6 by enhancement of synthesis and secretion of positive acute-phase plasma proteins. The ability of IL-6 to stimulate α_1_-antichymotrypsin (ACT) synthesis in Hep3B cells has been used as a bioassay for this cytokine [19]. When tested using fibroblast secreted IL-6 (calibrated in ng/mL by Western blotting against Coomassie Blue stained purified *E. coli*-derived IL-6), Hep3B cells were responsive by ACT secretion in the range 1–10 ng/mL (see Figure 1A,B in [19]). Using this assay, we estimated the concentration of IL-6 in serum in a patient with sepsis to be as high as 70 ng/mL, with that in CSF often >500 ng/mL (Table 1 in [19]). Western blotting of an immunoaffinity-column purified aliquot of serum from a patient with sepsis confirmed the valuation of 70 ng/mL of differentially modified IL-6 species (Figure 3 in [19]). Subsequent studies by us [22,23,70] confirmed concentrations of IL-6 in the range of 1–5 µg/mL in sera of cancer patients subjected to immunotherapy; such sera contained IL-6 in high molecular mass complexes as judged by Sephadex G-200 gel filtration [23,70] (Figure 6). Different mAb-based ELISAs gave different estimates of IL-6 depending on the mAbs selected for the design of the ELISA and the calibration standards used [70] (Figure 6). We note that May et al. [22] and Ndubuisi et al. [70] investigated ELISAs based on several different pairs of anti-IL-6 mAbs, including an mAb called “5IL6-H17” (Figure 6). This mAb is commercially available today as “Thermo-Pierce 5IL6” mAb and has proven to be the best mAb for the design of “panoptic” IL-6 ELISAs (i.e., assays that can see “total” IL-6 in serum-free as well as complexed IL-6) [71]. Indeed, using this panoptic ELISA, Chaturvedi et al. [71] confirmed that IL-6 in serum formed various high-molecular-mass complexes not visible using regular commercial ELISAs. 

Most investigators today utilize assorted commercial ELISA assays from their favorite biotech company in order to evaluate IL-6 levels in human body fluids. By and large these ELISAs do not detect “total” IL-6 in body fluids [70,71] (Figure 6). As a result, serum IL-6 values published in the literature reflect a range in the low pg/mL (see [70] and Table 2 in [71] for another comparative analysis)—an underestimate by several orders of magnitude [22,23,70,71]. Note that IL-6 at 10 pg or 100 pg/mL would have little or no detectable biological effect on Hep3B hepatocytes [19]. Chaturvedi et al. [66] acknowledge that none of the available ELISAs provide a formal evaluation of the absolute values of natural IL-6 in the circulation (in mass per volume). The key technical issue is that despite several decades having passed since the discovery of IL-6 in the 1980s, we still do not know how to measure IL-6 levels formally and reliably in human circulation in terms of absolute cytokine mass. Moreover, although IL-6 in complexes in the circulation has been largely ignored by clinical investigators, this issue becomes critical in cancer patients administered immunotherapy with “neutralizing” anti-IL-6 mAb (as in multiple myeloma and Castleman’s disease) and COVID-19. Not only does the administered anti-IL-6 mAb lead to the accumulation of high levels of IL-6 in circulation, but also a paradoxical enhancement of the biological activity of endogenous IL-6 [72,73].

## 7. Chaperone (Enhancing) Effect of Anti-IL-6 “Neutralizing” Antibodies on IL-6 In Vivo

It seems to be an article of faith today among investigators and physicians that treating patients with COVID-19 or cancer patients with anti-IL-6 mAb, which “neutralize” IL-6 bioactivity in cell culture, should have similar neutralizing effects in vivo [74]. Missing from many such studies is recognition of a salient property of IL-6 and anti-IL-6 Ab in vivo–the paradoxical enhancing (chaperone) effect of such Abs on the biological activity of IL-6 in vivo [73]. With the increasing use of chimeric antigen receptor T cell therapy (CAR-T), investigators have used anti-IL-6 mAb (siltuximab or tocilizumab) together with dexamethasone to manage the cytokine release syndrome (CRS) triggered by this therapy [75,76,77]. However, it is now clear that “one size does not fit all” and that many patients do not respond to anti-IL-6 mAb [76,77]. There is even confusion as to which IL-6 and sIL-6R ELISAs assays to use to measure the relevant cytokine (IL-6 and sIL-6R) in the serum; assays from different vendors give different results [75]. Again, missing from discussions of anti-IL-6 mAb therapy in the context of CAR-T-cell therapy is the concept that “neutralizing” antibodies might enhance biological effects in vivo [75,76,77].

In 1993, we reported that mice administered anti-IL-6 pAb, previously characterized to be a neutralizing Ab in assays in cell culture, injected into mice together with either LPS or rIL-6 paradoxically enhanced the levels of circulating fibrinogen [73]. This observation was unexpected because fibrinogen synthesis is strictly tied to IL-6 bioactivity, and the Ab we used was “neutralizing” in cell culture assays. Further experiments revealed that such “neutralizing” anti-IL-6 mAb prolonged not only the levels and duration of IL-6 in the circulation but also its biological activity. Finkelman et al. independently discovered this chaperone effect of anti-cytokine mAbs [78]. More generally, it is now recognized that many anti-cytokine “neutralizing” antibodies have an enhancing chaperone effect on biological activity in vivo [79,80,81]. The Ab: cytokine complexes appear to form a long-lived circulating reservoir from which the cytokine can percolate into target tissues and, paradoxically, elicit heightened biological responses [68]. In the case of anti-IL-6 mAb, van Zaanen et al. [72] marked paradoxical enhancement of circulating IL-6 in patients administered anti-IL-6 mAb [72]. Unraveling immunotherapy’s positive and negative aspects with anti-IL-6 mAb is likely to be disease- and patient-specific. This area of research (“the chaperone effects of neutralizing anti-cytokine mAbs”) remains minimally investigated. It is possible that the anti-IL-6-mAb administered to breast cancer patients undergoing CAR-T-cell therapy to manage their cytokine storm may inadvertently have unexpected metastasis-promoting effects. 

## 8. Conclusions

On the one hand, ample data accumulated over the last three decades to validate the involvement of IL-6 as an almost invariant presence at the host-tumor interface. Available data particularly highlight STAT3 activation in neoplastic cell elements consequent to local IL-6 effects as a key determinant of tumor progression and metastasis. On the other hand, there are significant gaps in our understanding of the transport and bioavailability of IL-6 in vivo. There is even difficulty in assessing absolute levels of IL-6 in the human circulation and target tissues. The chaperone effect of anti-IL-6 Abs adds a further layer of complexity to discussions of IL-6 bioactivity in vivo.

Both neoplastic and stromal cell elements in solid tumors express increased IL-6. Tumor-infiltrating immune cells and macrophages add a further contribution. In particular instances, sex-specific expression of IL-6 by macrophages accounts for the 3-5-fold increased prevalence of hepatocellular carcinoma in men compared to women. The ability of E2 to inhibit IL-6 production accounts for this sex-specific phenotype in the human disease and the DEN-induced mouse model of hepatocellular carcinoma.

A perplexing aspect of IL-6 biology in humans in vivo is the difficulty of deriving formally reliable absolute values of IL-6 in human circulation. Modern-day commercial ELISAs appear to underestimate IL-6 levels; nevertheless, most investigators simply accept such data at face value. A further conundrum is the paradoxical chaperone effect of so-called “neutralizing” Abs against IL-6—such Abs can prolong the presence of IL-6 in the circulation and enhance the bioavailability and bioactivity of this cytokine in vivo. Thus, there remain clear gaps in our understanding of the parameters that regulate the pathobiology and bioavailability of IL-6 in human circulation and tissues. 

In as much as IL-6 and STAT3 activation can enhance the proliferation of cancer cells in various solid tumors as well as hematopoietic malignancies, considerable effort is now focused on the development of STAT3 inhibitors as anti-cancer agents. The discovery that IL-6-activated STAT3 transcription factors form phase-separated biomolecular condensates in the cytoplasm and the nucleus provide a novel basis for targeting such STAT3 nanodroplet and condensate biochemistry in the cytoplasm and/or the nucleus for anti-cancer therapeutics. That 1,6-hexanediol, a membrane-permeable reagent that disrupts weak hydrophobic interactions, can disrupt such IL-6 activated STAT3 condensates (Figure 5) provides a proof of principle for this approach.

## Abbreviations

ACT	α_1_-antichymotrpsin
aFGF	acidic fibroblast growth factor
DEN	diethynitrosamine
E2	estradiol-17β
EMT	epithelial to mesenchymal transformation
ER	estradiol receptor
FRAP	fluorescence recovery after photobleaching
GR	glucocorticoid receptor
HCC	hepatocellular carcinoma
IL-6	interleukin-6
LPS	lipopolysaccharide
mAb	monoclonal antibody
MLO	membraneless organelle
MRE	multiple response element
SASP	senescence-associated secretory phenotype
